# Sustainable fisheries management through reliable restocking and stock enhancement evaluation with environmental DNA

**DOI:** 10.1038/s41598-023-38218-2

**Published:** 2023-07-12

**Authors:** Maslin Osathanunkul, Chatmongkon Suwannapoom

**Affiliations:** 1 Department of Biology, Faculty of Science, Chiang Mai University, Chiang Mai, 50200 Thailand; 2 School of Agriculture and Natural Resources, University of Phayao, Muang District, Phayao, 56000 Thailand

**Keywords:** Environmental biotechnology, Ichthyology

## Abstract

The practise of restocking and stock improvement as a means of managing fisheries and aquaculture has been widely used. However, it is difficult to claim that fish stocking is effective due to a number of challenges. One of those is the lack of suitable monitoring and assessment methods, although all assessment approaches have their strengths and weaknesses. If the full benefits of fisheries and their long-term sustainability are to be realised, it is necessary to examine the effectiveness of restocking and stock enhancement. Therefore, effective, rapid, and dependable monitoring techniques are necessary. In this study, we used an eDNA-based method to identify *G. cambodgiensis* at 14 sites throughout Thailand's restocking and stock enhancement programme. eDNA from this species was identified in water samples using quantitative polymerase chain reaction (qPCR) tests with primers and a probe specific to *G. cambodgiensis*. A successful stocking would show positive eDNA results in water samples collected from the studied sites. Only five of the studied sites returned positive eDNA readings, which could be considered a successful stocking. The locations that contained *G. cambodgiensis* eDNA were either confirmed to be natural habitats or were regularly stocked with a large number of hatchery fish. In this study, we demonstrated that eDNA is a reliable, fast and accurate alternative method for measuring stock improvement.

## Introduction

Freshwater fish are vital for communities, economies, and ecosystems, but unfortunately, the unprecedented level of pollution, habitat degradation, overfishing, and destructive fishing practices are having significant adverse effects on them. A recent global assessment of nearly 2500 rivers indicated that more than half of the studied sites have been heavily affected by human activities^1^. In addition, a third of global freshwater fish populations are threatened with extinction^2^, with 16 species already having been declared extinct in the year 2020 alone. Thailand is regarded internationally as one of the countries with high diversity of freshwater fish species; as many as 850 species have been reported, and about 10% of the world’s freshwater fish species live in Thailand^3^. Populations of freshwater fish are declining rapidly, and much of that decline is driven by human activities. When fish populations decline, overharvesting is often assumed to be the main reason behind it, as excessive fishing seems to be the most impactful human activity. Wild-caught freshwater fisheries in Thailand are mainly for sustenance^4^. They are especially critical for many poor and indigenous communities and sorely underestimated because the statistics commonly show country-level catches. Therefore, artisanal and subsistence fish catches are rarely documented.

Today, the Thai government is trying to reverse the loss of the freshwater fish population and put the inland fisheries back on a sustainable path. This includes an assessment of fish distribution throughout the main river basins in Thailand. Many of freshwater fish are in steep decline which is a strong indicator of what human have done. The Department of Fisheries of Thailand (DOF) recently launched a recovery plan for freshwater fish by launching a restoration and protection of rare, endemic, and endangered species project. Interestingly, one of the most undervalued and overlooked species, the stone lapping minnow (*Garra cambodgiensis*), has been added to the list of 39 fish species.

*G. cambodgiensis* is a tiny Cyprinidae fish species found in Southeast Asian stream and river systems, including the Mekong and Chao Phraya Basins^5–7^. They inhabit clean, swiftly moving water with pebbles. The species has a high economic importance for commercial fisheries and is recognised as a regional delicacy. *G. cambodgiensis* is one of the most widely consumed freshwater fish species in northern Thailand^8^. The fish is renowned for its flavour, especially the females with their roe intact. A fish can be consumed from head to tail, including its small bones. From June through August, the species migrates to rice fields and floodplains to spawn. During spawning season, the price of fish rises due to increased demand for fish carrying eggs; consequently, the overall catch of fish is rather large during this time. Under unrelenting pressure from habitat degradation, chemical contaminants, climate change, and notably overexploitation, *G. cambodgiensis* populations are presently in steep decline^9^.

One of the primary initiatives of the DOF's restoration and protection effort is restocking and stock enhancement. Restocking and stock enhancement has long been acknowledged as a beneficial fisheries management technique, even for overfished fisheries^10^. Restocking and stock enhancement may be carried out for a variety of reasons, including (but not limited to) the following: (i) increasing production of commercial species; (ii) aiding in the recovery of endangered species; (iii) establishing culture-based fisheries; (iv) enhancing or supplementing self-recruiting populations; and (v) introducing restoration practises in areas where the capacity to expand stocks naturally has been lost due to the devastation of spawning grounds or the loss of ecosystem connectivity^11^. However, the effectiveness and correct use of these programs are still without a scientific basis^12^. Mathematical models have been used to analyse the effect of refilling grazing fish as a restoration technique, and to analyse the financial outcomes of the restocking operation^13^. Genetic data has also been used to inform decisions on restocking and other aspects of management for unusual fish populations^14^. Nonetheless, it is difficult to claim the successful stocking of any impoverished species due to numerous obstacles, including the production of cost-effective juveniles, the development of adequate release tactics, and the availability of suitable monitoring and evaluation techniques^10,15^. The success of stock enhancement must be evaluated in order to maximise the potential benefits of fisheries and ensure their sustainability. In most cases, monitoring and evaluation efforts are insufficient, therefore a proper assessment of programme efficacy cannot be conducted^16^. Development of reasonable and effective monitoring techniques that are non-intrusive and will not make any harms depleted species (and make matters worse) is therefore crucial.

In addition, functional monitoring is an essential component of fisheries management because it enables the evaluation of the health and sustainability of fish populations. One of the primary obstacles in Asia is limited management and research capacity^17^. This lack of capacity has discouraged a number of sophisticated modelling and monitoring approaches for Asian fisheries, which could significantly enhance the accuracy and efficacy of monitoring efforts. Despite these challenges, there are still efforts being made to improve functional monitoring in Asia. Community-based fisheries management (CBFM) approaches have been proposed^18^ to resolve the lack of management and research capacity in Asian fisheries. Building trust, participatory approaches, effective leadership, and capacity building are crucial components for achieving sustainability goals in Southeast Asian fisheries^19^. Nevertheless, monitoring fishing activities based on landings data and scientific surveys continues to be a difficult undertaking^20^. The use of environmental DNA (eDNA)-based detection is a possible remedy to this problem. To implement eDNA-based detection in Asian fisheries, however, requires additional research and development. In recent years, there has been a rise in interest in monitoring or surveying techniques that employ eDNA. eDNA-based biomonitoring has been found to be more sensitive than traditional survey methods, which can be time-consuming and costly^21^. In aquatic environments, eDNA analysis has been found to be an economical, efficient, and sensitive tool^22^. eDNA methods have been used to monitor aquatic vertebrates in stream habitats^23^, detect invasive or endangered aquatic species^24^, examine species diversity and biomass^24^, and even detect terrestrial mammals from forest pond water^25^. Overall, eDNA-based methods have shown great promise for monitoring aquatic organisms and have the potential to outcompete conventional methods in terms of higher detection sensitivity, lowered sampling effort, and associated survey costs^26^.

Although eDNA is a potent monitoring instrument for aquatic ecosystems, its efficacy can vary depending on the type of water system. Studies have demonstrated that eDNA is more effective in confined freshwater systems like ponds, rivers, and lakes than in open oceans^22,27–29^. Due to reduced water flow and fewer external factors that can degrade the DNA, eDNA is more likely to persist in freshwater environments^27–29^. It has been discovered that salinity degrades eDNA faster than freshwater^27,28^. Moreover, it has been demonstrated that eDNA degrades more rapidly in environments influenced by land than in environments influenced by the ocean^28^. Overall, eDNA is a reliable tool for detecting fish communities in dynamic freshwater habitats, and its application in biomonitoring is becoming standardised^30,31^. However, additional habitat-specific assessments are required to completely comprehend the efficacy of eDNA in various aquatic ecosystems^32^.

As previously mentioned, *G. cambodgiensis* is now included in the DOF's restoration and protection project, using stock enhancement as the primary strategy. However, conventional monitoring approaches are often time-consuming, difficult to estimate on a large scale, and necessitate a large sampling effort with the potential to harm the target species. The use of eDNA monitoring to assess the performance of conservation and restoration management initiatives is currently widespread. eDNA detection was used to measure the efficacy and success of large-scale dam removal^33,34^, river restoration^35^, and invasive species eradication efforts^36–38^. The ever-increasing study of eDNA demonstrates that the detection of eDNA is useful for evaluating the effectiveness of restoration or management programmes. In this study, an eDNA-based survey was therefore used to monitor the presence of *G. cambodgiensis* where hatchery release programmes have been implemented. The results will be useful for the evaluation of the programme as well as the identification of the key natural habitat of the fish.

## Results

### eDNA detection

A total of 42 eDNA samples (triplicate of 14 samples from each site) were collected and analysed at 14 sites where *G. cambodgiensis* hatchery release programmes have been implemented (Table 1). *G. cambodgiensis* eDNA was only discovered in water samples from five locations: F, G, I, L, and M. In samples with positive detections, the concentration of *G. cambodgiensis* eDNA ranged from 3.3 to 531.9 copies per millilitre (Table 2). Two sampling sites, F (531.9 copies/mL) and I (458.4 copies/mL), exhibited a high eDNA content. In the three remaining positive sites, G (4.7 copies/mL), L (5.3 copies/mL), and M (3.3 copies/mL), low concentrations were observed. The eDNA concentration of all qPCR-positive samples is shown in Table S1. There was no *G. cambodgiensis* eDNA found in the remaining sites (A-E, H, J-K, and N).

**Table 1:** Locations of sampling sites and number of *G. cambodgiensis* released annually at each location.

Site	Date of water collection	Location	Coordination	Record of stocking in the past	Number of released fish in each year				
					2016	2018	2019	2020	2021
A	6 April 2022	Chedi Chai, Pua District, Nan	19.165100, 100.812810	Yes	7500	–	–	–	–
B	6 April 2022	Nai Wiang, Mueang Nan District, Nan	18.782063, 100.785557	Yes	–	–	–	10,000	–
C	7 April 2022	Khueng, Wiang Sa District, Nan	18.550198, 100.759647	Yes	–	–	–	10,000	–
D	25 March 2022	Na Thanung, Na Muen District, Nan	18.053728, 100.679651	Yes	–	–	–	10,000	–
E	25 March 2022	Pha Tong, Tha Wang Pha District, Nan	19.179601, 100.769706	Yes	–	–	10,000	–	10,000
F	6 April 2022	Mae Charim, Mae Charim District, Nan	18.846309, 101.028922	Yes	–	–	10,000	–	10,000
G	7 April 2022	Phu Fa, Bo Kluea District, Nan	19.003138, 101.214144	Yes	–	10,000	10,000	15,000	15,000
H	25 March 2022	Pa Kha, Tha Wang Pha District, Nan	19.093918, 100.583726	Yes	–	10,000	10,000	10,000	10,000
I	21 March 2022	Phu Kha, Pua District, Nan	19.254929, 101.071406	Yes	–	10,000	10,000	10,000	10,000
J	22 March 2022	Mo Mueang, Mae Charim District, Nan	18.688047, 101.027448	No	–	–	–	–	5000
K	3 April 2022	Na Noi, Na Noi District, Nan	18.315572, 100.686488	No	–	–	–	–	5000
L	3 April 2022	Na Rai Luang, Song Khwae District, Nan	19.304048, 100.715161	No	–	–	–	–	10,000
M	25 March 2022	Thuem Tong, Mueang Nan District, Nan	18.800605, 100.700825	No	–	–	–	–	5000
N	3 April 2022	Sisaket, Na Noi District, Nan	18.360913, 100.728651	No	–	–	–	–	3000

**Table 2:** eDNA detection results for each site. At each site, three samples. Each extracted sample was then used in six qPCR replicates. All qPCR results were divided into three categories: (i) positive, + (with quantifiable eDNA concentration), (ii) below limit of quantification, *bq* (Cq = 37.72—44.99), and (iii) non-detect, *nd* (Cq 45 or No amplification). The concentration of eDNA was expressed as copies/mL.

Site	eDNA detection*	eDNA concentration (copies/mL)	
		Mean	SD
A	*nd*	–	–
B	*bq*	–	–
C	*nd*	–	–
D	*nd*	–	–
E	*bq*	–	–
F	+	531.3	8.79
G	+	4.7	0.42
H	*nd*	–	–
I	+	458.4	10.15
J	*nd*	–	–
K	*nd*	–	–
L	+	5.3	3.15
M	+	3.3	0.11
N	*nd*	–	–

### Data analysis

In this study, three water samples were collected from each of 14 sites located in Nan, Thailand. Samples were taken from different spots at each sampling site due to accessibility while we tried to attain representative spatial coverage in a reasonable amount of time. Detections and no detections of *G. cambodgiensis* eDNA were assessed using qPCR in each of six subsamples extracted from each water sample. Eight covariates of each sampling sites were recorded in which seven of them were used to created habitat suitability score or HSS (Tables 3 and 4).

**Table 3:** Parameters and criteria used for scoring habitat suitability. 1 indicates that the value is preferable for the species, while 0 indicates that the value is outside of the species' flavoured range.

Parameter	Criteria	Score
Temperature	20 °C–26 °C	1
	< 20 °C or > 26 °C	0
Rocky substrates	Presence	1
	Absence	0
Moving water	Yes	1
	No	0
Waterbody size	Medium or Small	1
	Large	0
Biofilm	Presence	1
	Absence	0
pH	6–7.5	1
	< 6 or > 7.5	0
TDS	≤ 215 ppm	1
	> 215 ppm	0

**Table 4:** Habitat suitability score (HSS) of each sampling site. Each environmental factor was rated based on the preference of the examined species, and the scores were combined to form HSS. Turbidity was evaluated indirectly via filtration time and was not factored into the HSS score.

Site	Temperature		Rocky substrates		Moving water		Waterbody size		Biofilm		pH		TDS		Habitat suitability score (HSS)	Turbidity (TUR)
	°C	Score	Presence/absence	Score	Yes/no	Score	Large/medium/small	Score	Yes/no	Score	–	Score	ppm	Score		
A	26.8	0	Absence	0	Yes	1	Large	0	No	0	7.28	1	202	0	**2**	+ + + + +
B	29.6	0	Absence	0	Yes	1	Large	0	No	0	7.28	1	177	0	**2**	+ + + + +
C	27.1	0	Absence	0	Yes	1	Large	0	No	0	7.28	1	169	0	**2**	+ + + + +
D	28.5	0	Absence	0	Yes	1	Large	0	No	0	6.91	1	169	0	**2**	+ + + + +
E	27.3	0	Absence	0	Yes	1	Large	1	No	0	7.42	1	198	0	**3**	+ + + + +
F	32.6	1	Presence	1	Yes	1	Medium	1	Yes	1	6.73	1	176	1	**7**	+ +
G	24.9	1	Presence	1	Yes	1	Medium	1	No	0	7.47	1	216	1	**6**	+ + + + +
H	24	1	Absence	0	Yes	1	Medium	1	No	0	7.08	1	75.3	0	**4**	+ + + + +
I	24.5	1	Presence	1	Yes	1	Small	1	No	0	7.18	1	81.3	1	**6**	+ + +
J	26.4	1	Absence	0	Yes	1	Large	0	No	0	7.32	1	183	1	**4**	+ + + + +
K	28	0	Absence	0	No	0	Medium	1	Yes	1	6.94	1	228	0	**3**	+ + + +
L	25	1	Presence	1	Yes	1	Large	0	No	0	7.39	1	161	1	**5**	+ + + + +
M	27.3	0	Presence	1	No	0	Medium	1	Yes	1	6.76	1	36.8	1	**5**	+ + +
N	30.1	0	Absence	0	No	0	Small	1	Yes	1	7.13	1	461	0	**3**	+ + + +

To test if probability of detection differs as a function of HSS or TUR (turbidity), the EDNAOCCUPANCY R package^39^ was used to model probabilities of eDNA detection. This package fits Bayesian, multi-scale occupancy models to our data, which included three, nested levels of sampling: sampling site (A-N), replicated water samples collected from each site (sample1-sample3), and subsamples (six qPCR technical replicates) of each water sample. In a multiscale occupancy model HSS was set as a covariate of eDNA occurrence at sampling sites, and HSS and TUR as a covariate of eDNA occurrence in both samples and qPCR replicates. This was the occupancy model with the best support according to the model-selection criteria posterior predictive loss criterion (PPLC) and the widely applicable information criterion (WAIC). The posterior medians of the model’s formal parameters are shown in Figure S1 and the derived parameters of the models (probabilities of eDNA site occupancy, sample occupancy and detection) were also shown in Figure S2. The results suggest that (i) the occurrence of *G. cambodgiensis* eDNA is higher at sampling site with high value of HSS, (ii) the occurrence of *G. cambodgiensis* eDNA in samples is unaffected by HSS and TUR and (iii) the detection of *G. cambodgiensis* eDNA in PCR replicates increased with HSS but decreases with TUR.

In the Rshiny app^40^, Bayesian models integrating false-positive and false-negative errors were also used to predict false presence. The posterior inclusion probabilities (PIPs) for the probability of occupancy can be used to determine how effective each covariate is as a predictor for the corresponding parameter. A high PIP (above the threshold of 0.5) indicates greater support for a covariate's predictive ability. In this instance, HSS and TUR are significant predictors of the likelihood that a site is inhabited by *G. cambodgiensis* (Figure S3).

The application then generates the posterior probability of species absence based on the number of positive qPCR replicates. The results indicated that the posterior conditional probability of species absence is greater than 80% if there are two or fewer positive qPCR replicates but declines precipitously to approximately 4.7% at the final qPCR replicate in this study (Figure S4). In addition, the posterior probability of positive qPCR replicates dependent on the presence of a species was calculated, revealing that the posterior probability of zero qPCR positives given the presence of a species is just under 7%, decreasing for qPCR replicate = 1, 2, and increasing to nearly 50% for six replicates (Figure S4). According to Diana et al. (2021)^40^, this could be a result of observation error in stage 1: the first peak at 0 is the result of a stage 1 false negative observation, while the second peak is the result of a stage 1 true positive observation.

## Discussion

According to a number of studies, the pandemic caused a major decrease in the demand for fresh fish products in a number of regions, which has spurred local exploitation due to a growing need for fish sourced locally^41,42^. In Asia, stocking and introduction of fish have been frequently utilised to counteract losses to native stocks and decreases in commercial catches, such as stocking programmes for *Catlocarpio siamensis*, *Pangasianodon gigas*, *Probarbus labeamajor*, and *P. labeaminor*^43^. Stocking of indigenous species in vast bodies of water has recently gained popularity in various Asian countries, such as Thailand^44^. Stock enhancement of inland waters has been practised since the early 1950s in Thailand, beginning with the stocking of *Trichogaster pectoralis* and *Oreochromis mossambicus*^45^. Later, there was a growing push towards the conservation or repopulation of economically significant indigenous species such as *Pangasius hypophthalmus*, *Pangasianodon gigas*, *Probarbus jullieni*, *Chitala chitala*, and *Osphronemus gourami*^45^. Recently, Thailand's Department of Fisheries (DOF) initiated a restoration and protection effort, and *G. cambodgiensis* was added to a list of 39 fish species. Around 1 million fingerlings of *G. cambodgiensis* were stocked in Thai rivers in Northern areas in one or more years between 2016 and 2021.

Fish stock enhancement into a new water body can be established for a variety of purposes, including (1) improving commercial or subsistence fisheries, (2) increasing food supply, and (3) restoring or recovering uncommon, threatened, or endangered fish species^10^. Fish monitoring data and information could be utilised to determine the success or failure of a stocking effort. As a result, monitoring efforts must be dependable and efficient. Fish monitoring or surveying using eDNA has been demonstrated to be one such strategy. In numerous cases, including endangered species, eDNA-based monitoring has been shown to be superior to traditional catch-based evaluations^46^. In this case, an eDNA-based detection appears to be an appropriate tool for assessing *G. cambodgiensis* stock enhancement in Thailand. The *G. cambodgiensis* are so little such that capture-based assessment would rely on a choice of fishing gears. Although there is a wide range of fishing gear available. Gillnets and/or trammelnets, seinenets, castnets, and liftnets are common types of gear used in stocked improved fisheries^31^. The catch efficiency of various fishing gears would differ. Numerous studies have also found that traditional fish survey methods may be biased towards various features such as species, size, season, and habitat^47^. Furthermore, monitoring should be done on a continual basis, and the eDNA-based technique is ideal for this because it is cost-effective particular for survey in high-diversity areas^48^.

Many factors, including predators, food availability, water body carrying capacity, and temperature, can affect the survival, growth, dispersal, and reproduction of hatchery-reared fish, thus they must be taken into account^49^. As stated in a number of prior articles, the failure of stocking programmes could result from a failure to examine or evaluate ecological and biological elements in their planning^50,51^. This could be one of the reasons why *G. cambodgiensis* eDNA was not detected in the majority of the water bodies (9 out of 14 sites) examined in this investigation, indicating that the fish were absent. In addition, only three sites were found eligible for stock enhancement of *G. cambodgiensis* in this study (Table 4). Prior to commencing the programme, it is unquestionably required to examine the fish release size, release season, release habitat, and release quantity in order to prevent its failure^49^. Our conclusion is therefore a significant piece of evidence demonstrating that stock enhancement for fish conservation cannot be successful without adequate attempts to analyse the released habitats.

In addition to habitat concerns, a paucity of fertilised eggs and/or larvae can be caused by a shortage of breeding adults or a general failure to successfully reproduce. Habitat deterioration or abnormal weather conditions are typically implicated in the second situation, whereas overfishing is typically implicated in the first^52^. The natural recruitment and production of fish in small waterbodies are typically insufficient to support a major fishery. Due to its tiny size, the body of water contains few areas that cannot be easily fished; consequently, the native fish population is quite susceptible to overfishing, despite the body of water's potentially high productivity. Hence, fisheries development in small waterbodies is typically conducted in tandem with a regular stock enhancement programme^44^. The results presented here indicate that *G. cambodgiensis* eDNA can only be found at sites with regular stock or sites with a significant number of released hatchery fish (F, G, and I). Interestingly, small waterbodies are typically managed by individuals or organised groups of individuals. They determine who has access to the water and how much fishing is done, and in some instances, they take steps to improve the stock^44^. This may be one of the reasons for the positive eDNA results observed at sampling locations L and M in this investigation, as both sites were administered by locals, fishing was restricted, and the fish in the bodies of water were routinely fed (personal communications). This is an excellent example of follow-up management measures that could continuously maintain the effects of restocking in the long term and would therefore benefit the programme.

Moreover, the genetic marker used in this study does not appear to be specific to hatchery fish, so it cannot differentiate between eDNA secreted by hatchery fish and eDNA shed by naturally occurring fish. The development of a genetic marker specific to hatchery fish that can be used to locate introduced fish would be useful for quantifying the contribution of stocking programmes to the fish population at a particular location based on the prevalence of the marker. However, the primary purpose of the *G. cambodgiensis* stock enhancement in Thailand was to conserve the species. So that the detection of *G. cambodgiensis* eDNA in any of the programme's sites would likely deem a success. Although, the study sites J-N have never been stocked in the past, *G. cambodgiensis* eDNA was only detected at sites L and M, suggesting that these sites were more suitable for the species.

Limnological processes in aquatic systems can significantly influence the degradation and transport of environmental DNA (eDNA) within the system. eDNA can be transported over long distances in stream and river systems, resulting in its detection at downstream sites^53^. The presence of benthic biofilm in streams can strongly influence the degradation of eDNA^54^, but the role of biofilms in natural systems is still not well understood. The degradation of eDNA in lotic environments is typically assumed to be a linear decline with increasing distance from the source, barring major changes in hydrology^55^. However, the transport and deposition of eDNA can be influenced by various factors, including the physical properties of the stream, such as morphology and hydrodynamics^56^. The spatial distribution of eDNA in aquatic systems can also be impacted by the bottom substrate of the stream^57^. Additionally, the persistence of eDNA can be affected by environmental factors such as acidity^58^. While laboratory studies have investigated the effects of some environmental factors on eDNA persistence and transport, field studies comparing the spatial distribution of eDNA with expectations based on prior knowledge of organisms' distributions are critical to developing a working understanding of eDNA in the real world^59^. The downstream decrease in eDNA is comparable to that observed for fine particulate organic matter and is highly dependent on the local hydraulic characteristics^60^. Understanding the intricate interactions between limnological processes and eDNA transport and degradation is therefore one of the most important factors in accurately detecting and monitoring aquatic species in natural systems.

As the possibility that the eDNA found at a site were transported from elsewhere or the eDNA found at a site came from a naturally occurring population of fish that inhabit the site could be a source of error that can lead to false negative or false positive results^55^. Another source of false negatives is the failure to capture the genetic marker at a site, which can occur due to factors such as low DNA concentration or degradation^61^. To account for false positive and false negative errors in eDNA surveys, occupancy modelling has been proposed as a useful tool^62^. This approach allows for the estimation of detection probabilities and the detection of false negatives, which can be used to adjust occupancy estimates. Additionally, analyses of primer amplification bias using tissue from target species or in silico tests of primer specificity can inform appropriate genetic marker selection^63^. Determining the precise source of eDNA in flowing systems is challenging due to the combined effect of downstream transport and eDNA degradation, which alter eDNA concentration in the water column after it is released from an organism^64^. Furthermore, eDNA studies have shown contrasting results related to its detection scale and the number of species identified compared to other survey methods^65^. In order to obtain high-quality eDNA, it is recommended to use 1 or 2 L surface water collection and eDNA capture on 0.7-μm glass fiber filters followed by extraction with a DNeasy Blood and Tissue Kit or PowerWater DNA Isolation Kit^66^. In addition, the use of droplet digital polymerase chain reaction (ddPCR) and the use of EMM in real-time PCR assays have the potential to decrease false negative eDNA detection rates without increasing sampling effort^67^. It is also important to understand the influence laboratory methods such as DNA extraction and PCR strategies have on detection probability^68^. In conclusion, while eDNA-based detection is a promising tool for monitoring aquatic biodiversity, it is important to consider potential sources of error and use appropriate methods to account for them. Occupancy modelling and a priori analyses of primer amplification bias can help to address false positive and false negative errors, while careful consideration of sampling and laboratory methods can improve detection probability and accuracy.

## Conclusions

Due to its non-invasive nature and high sensitivity, eDNA-based detection was selected to monitor and evaluate the *G. cambodgiensis* stock enhancement in Thailand. Our research focuses on restocking effects based on eDNA concentration, and we believe it is essential to recognise that habitat suitability is determined by a number of ecological factors. Additional statistical analyses, such as regression or additive modelling, would provide a more complete comprehension of the success of the stocking programme. There are potential sources of error that can lead to false negative or false positive results, and it is essential to consider these potential sources of error and account for them using appropriate methods. In this study, occupancy modelling was used to analyse the data, and primer amplification bias was performed both in silico and in vitro, which we believe can aid in addressing false positive and false negative errors. In addition, the sampling strategy and laboratory procedures were meticulously planned, which is likely to increase the probability and precision of eDNA detection. As a result of investigating the relationships between eDNA concentration and other environmental factors, future restocking projects can be designed more efficiently, taking into consideration the variables that have the greatest impact on the persistence and fitness of the species.

## Material and methods

### Ethics statement

Ethical approval for this study was obtained from the Institute of Animals for Scientific Purposes Development (IAD), University of Phayao (protocol number: 610104004).

### Water sampling and DNA extraction

**Figure 1:**
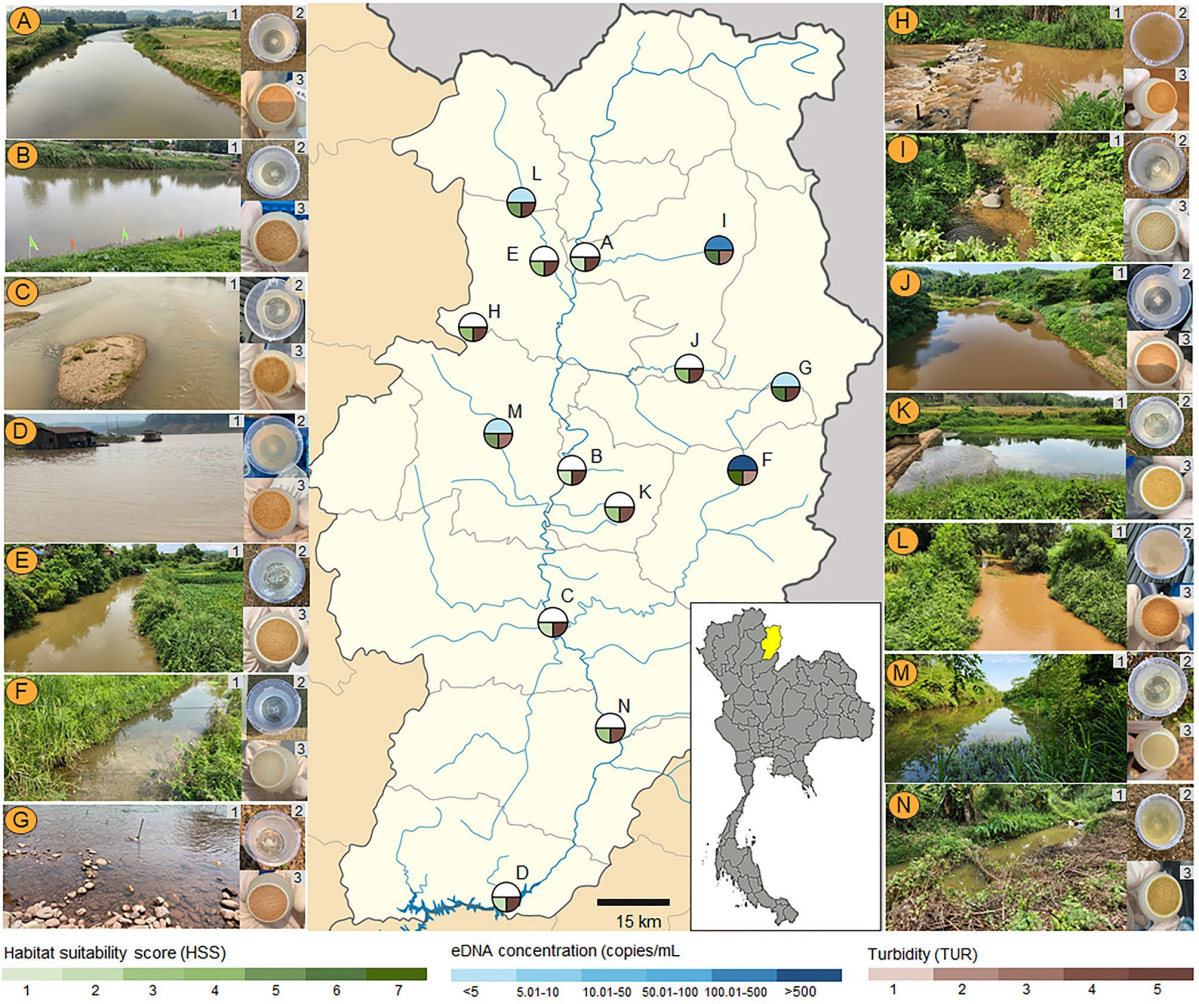
Pictures of (1) sampling sites, (2) water samples collected from each site, and (3) filters. At each sampling site on the map, circles of varying colours represent the eDNA concentration, habitat suitability score (HSS), and turbidity (TUR).

Water sampling and DNA extraction were conducted according to Osathanunkul and Minamoto^69^. Water samples were collected from the surface at 14 sites in Nan province, Thailand (Table 1 and Fig. 1). According to data supplied by the Department of Fisheries, fish were released only once at nine sites: site A (year 2016), sites B-D (year 2020), and sites J-N. (year 2021). While fish were released twice at two sites during the course of the programme, sites E and F (year 2019 and 2021). From 2018 to 2021, the fish were released at the three remaining sites (G-I) for four consecutive years (Table 1).

At each sampling site, 300 mL of water were immediately filtered in the field using a BD Luer-Lok™ syringe and a glass fibre 0.7 µm filter (Whatman GF/F). Three samples (3 filters for each sample) were collected at each site. Samples were taken from different spots at each sampling site due to accessibility while we tried to attain representative spatial coverage in a reasonable amount of time. As a field-negative filtration control, 300 mL of distilled water was filtered at each site in the same manner as water samples. Hence, this technique was executed at each location with a total of 18 filters (3 filters and 3 negative filters for each sample). Before extraction, all filters were placed in 1.5-mL microcentrifuge tubes and stored in a polystyrene box containing dry ice before being moved to a 20 °C freezer. Using the DNeasy Blood and Tissue Kit (Qiagen, Hilden, Germany) with the protocol from Osathanunkul and Minamoto^69^, all samples were extracted within 48 h after collection. Three filters of each sample were added into the same extraction column (300 mL of water filtrated through each filter, so combining the three makes 900 mL as the total volume for each sample. Samples from all 14 locations (N = 42) were eluted twice in 50 µL of TE buffer and then stored at 20 °C. To eliminate any PCR inhibitors, the samples were processed with the OneStep PCR Inhibitor Removal Kit (Zymo Research). The inhibition of the PCRs of water samples was performed using primers and probes targeting the 16S rRNA of jellyfish species, *Chiropsoides buitendijki*, a marine species which does not inhabit the streams (forward primer: 5′-CCCCAATCGAAATTAAGTTAGCC-3′; reverse primer: 5′-CACAGGTAGAGTGGAGAAATAGAG-3′; probe: 5′-FAM-GTGAAGACGCAGCTTTGTCT-TAMRA-3′). The oligo synthesis of *C. buitendijki* (1.5 × 10^2^ copies) was added to the samples (gBlocks™ Gene Fragments, IDT). The average of ΔCq values from the internal controls of all samples were less than 3, which was lower than the inhibition criteria (Cq shift of ≥ 3 cycles was an indication of inhibition). Therefore, PCR inhibition was not likely to occur in all samples.

### Developing qPCR assay

The qPCR tests were conducted in accordance with Osathanunkul and Suwannapoom^70^. GenBank Primer-BLAST was originally used for in silico analysis to determine the primers-probe's specificity. Specificity was then tested by doing quantitative polymerase chain reaction (qPCR) tests on DNA extracted from mucus. Separate laboratories were used for filter-based eDNA extraction and qPCR testing to reduce the risk of cross-contamination. Before being used, all tools, materials, and workspaces were sterilised using bleaching and 70% EtOH spraying, respectively. Three individuals of *G. cambodgiensis* and one individual of each non-target fish species or co-occurring species had total DNA extracted from their mucus samples using the Qiagen DNeasy Blood and Tissue Kit (Qiagen, Valencia, CA). Both the extracted DNA and synthesised fragments were employed as templates in the qPCR analysis. Primers and probe designed specifically for *G. cambodgiensis* were used in the qPCR experiments, which amplified a 109-bp region of the mitochondrial cytochrome C oxidase subunit I (COI) gene^70^. Forward primer GarraCamCOI-F250 has the sequence 5′-GGGTTTGGAAACTGGCTC-3′, reverse primer GarraCamCOI-R337 has the sequence 5′-ATAATAGCAGGAATGATGGTGG-3′, and probe GarraCamCOI-P287 has the sequence FAM 5′-CCCCCGACATGGCATTTC-3′ MGB. Species-specific primers were used in qPCR, and their specificity was evaluated by using them on non-target species or co-occurring species in the same geographic range.

### qPCR of water samples

qPCR was performed on water samples in a manner similar to Osathanunkul^71^ with a few minor modifications. Six qPCR assays were conducted on each water sample. For each individual 20 μl qPCR reaction containing 10.0 µL of 2 × TaqMan Environmental Master Mix 2.0 (Thermo Fisher Scientific), 2.0 µL of DNA template, 900 nM each of the F/R primers, and 125 nM of the probe were prepared in triplicate. No template controls (NTC) with all qPCR reagents but no template (three replicates) were run in parallel to monitor potential contamination. As positive and negative controls, the qPCR analysis used water samples from ponds of farmed fish that contained and lacked *G. cambodgiensis*. The temperature gradient assay was used to optimise the qPCR conditions. The qPCR program consisted of 95 °C for 10 min, followed by 50 cycles of 95 °C for 15 s, and 60 °C for 1 min. All qPCR reactions were carried out using Rotor-Gene Q MDx 5plex (Qiagen Valencia, CA). qPCR of water samples was performed similarly to the mucus DNA tests. qPCR reactions for each water sample were run in triplicate. To confirm target species amplification, positive eDNA detections from each sampling point were sent for sequencing.

Standard dilution series of synthesised target gene fragments with known copy numbers were used to determine the Limit of detection (LOD) and limit of quantification (LOQ). Twelve technical replicates of a dilution series ranging from 1.5 × 10^–1^ to 1.5 × 10^5^ copies per qPCR tube were generated and used as quantification standards. Using a 2 μL standard dilution series of each synthesised target, a standard curve for *G. cambodgiensis* was constructed (y = -3.5789 + 42.634, R2 = 0.9912, efficiency = 90.29%). Klymus et al. (2020) have a published R script that was used here to determine the LOD and LOQ which found to be 25.44 copies/reactions for both LOD and LOQ. Each sample's concentration was determined using the synthesised target gene standard curve (Integrated DNA Technologies Pte. Ltd., Singapore), and results were reported as copies/mL. Positive detections of *G. cambodgiensis* eDNA were defined as Cq values of 37.71 or lower. All qPCR findings were categorised into three groups as described in Osathanunkul and Suwannapoom^70^. Briefly, positive, + (with quantifiable eDNA concentration), below limit of quantification, *bq* (Cq = 37.72–44.99), and non-detect, *nd* (Cq 45 or No amplification).

### Data analysis

Since eDNA is heterogeneously disseminated in water, the detection probability was estimated with occupancy models EDNAOCCUPANCY in R using Markov Chain Monte Carlo (MCMC) methods of maximum-likelihood^39^. *G. cambodgiensis* detection probabilities and the conditional probability of *G. cambodgiensis* DNA in a field sample or qPCR replicate can be estimated. The nested sampling design for eDNA sampling consisted of location (sampling sites A–N), field sample (sample1–sample3), and qPCR repetition (6 replicates). In the model, eDNA is present at a particular location, in replicate field samples, and in replicate qPCR reactions.

To further evaluate potential false-negative and false-positive errors resulting from field sample collection and lab. The Bayesian framework of Griffin et al. (2020)^72^, implemented in a Rshiny app^40^, was used with the default setting. The analysis can determine the probability of species presence at a site (ψ), a sample with positive DNA from a site with presence of target species (θ_11_), a sample with positive DNA from a site without the target (θ_10_), a positive qPCR replicate of a sample with target species DNA (*p*_11_), and a positive qPCR replicate of a sample without target species DNA (*p*_10_). False-negative probabilities of field and lab are defined as 1- θ_11_ and 1- *p*_11_, respectively.

In addition to collecting water samples, several covariates known to be predictive of *G. cambodgiensis* occurrence were recorded. Tables 3 and 4 list the covariates used to calculate the habitat suitability score (HSS), along with the scoring criteria. Apart from seven covariates used for scoring habitat suitability, turbidity (indirectly assessed using filtering time) was recorded and used in the data analysis (EDNAOCCUPANCY and Rshiny app).

## Supplementary Information

Supplementary Information.

## Data Availability

The datasets generated and/or analyzed during the current study are available in the GenBank repository, [Accession Numbers were provided in Table S2].
