# Supplementary material for: Sustainable fisheries management through reliable restocking and stock enhancement evaluation with environmental DNA
Source: Sci Rep. 2023 Jul 12;13:11297. doi: 10.1038/s41598-023-38218-2 (PMC10338448; doi:10.1038/s41598-023-38218-2)
Supplement: Supplementary file 1 — Supplementary Information. [file 41598_2023_38218_MOESM1_ESM.pdf]

Supplementary Information for

Sustainable Fisheries Management through Reliable Restocking and Stock Enhancement Evaluation with environmental DNA

Maslin Osathanunkul[1]* and Chatmongkon Suwannapoom[2]*

[1]Department of Biology, Faculty of Science, Chiang Mai University, Chiang Mai, Thailand

[2]School of Agriculture and Natural Resources, University of Phayao, Muang District, Phayao, Thailand

This PDF file includes:

Figures S1 to S4

Table S1 to S2

Bayesian estimates of model parameters

	Mean	50%	2.5%	97.5%
beta.(Intercept)	-0.220	-0.144	-0.710	-0.144
beta.HSS	1.772	1.419	1.419	3.379
alpha.(Intercept)	6.702	6.702	6.702	6.702
alpha.HSS	0.000	0.000	0.000	0.000
alpha.TUR	0.000	0.000	0.000	0.000
delta.(Intercept)	-0.303	-0.301	-0.944	0.334
delta.HSS	0.990	0.978	0.297	1.736
delta.TUR	-0.449	-0.446	-0.779	-0.131

Monte Carlo SE of Bayesian estimates

	Mean	50%	2.5%	97.5%
beta.(Intercept)	0.0502	0.0019	0.0282	0.0019
beta.HSS	0.2399	0.0376	0.0376	0.0000
alpha.(Intercept)	0.0000	0.0000	0.0000	0.0000
alpha.HSS	0.0000	0.0000	0.0000	0.0000
alpha.TUR	0.0000	0.0000	0.0000	0.0000
delta.(Intercept)	0.0047	0.0059	0.0090	0.0130
delta.HSS	0.0051	0.0065	0.0116	0.0131
delta.TUR	0.0022	0.0029	0.0062	0.0052

NULL

Figure S1. Estimating posterior summaries of the model's formal parameters, when fitting a multiscale occupancy model that uses habitat suitability score (HSS) as a covariate of eDNA occurrence at sampling sites, and HSS and turbidity (TUR) as a covariate of eDNA occurrence in samples and qPCR replicates.

	psi	theta	p
A	0.04246015	1	0.04766155
B	0.04246015	1	0.04766155
C	0.04246015	1	0.04766155
D	0.04246015	1	0.04766155
E	0.26842030	1	0.18180400
F	0.99651299	1	0.99635622
G	0.94440371	1	0.73317486
H	0.68709367	1	0.44256623
I	0.94440371	1	0.93199298
J	0.26842030	1	0.18180400
K	0.26842030	1	0.31852408
L	0.68709367	1	0.44256623
M	0.68709367	1	0.76944410
N	0.26842030	1	0.31852408

Figure S2. The probability of eDNA occurrence in sampling sites was assumed to be constant (psi), the conditional probability of eDNA occurrence in samples was assumed to be a function of the HSS (theta), and the conditional probability of eDNA detection was assumed to be constant (p).

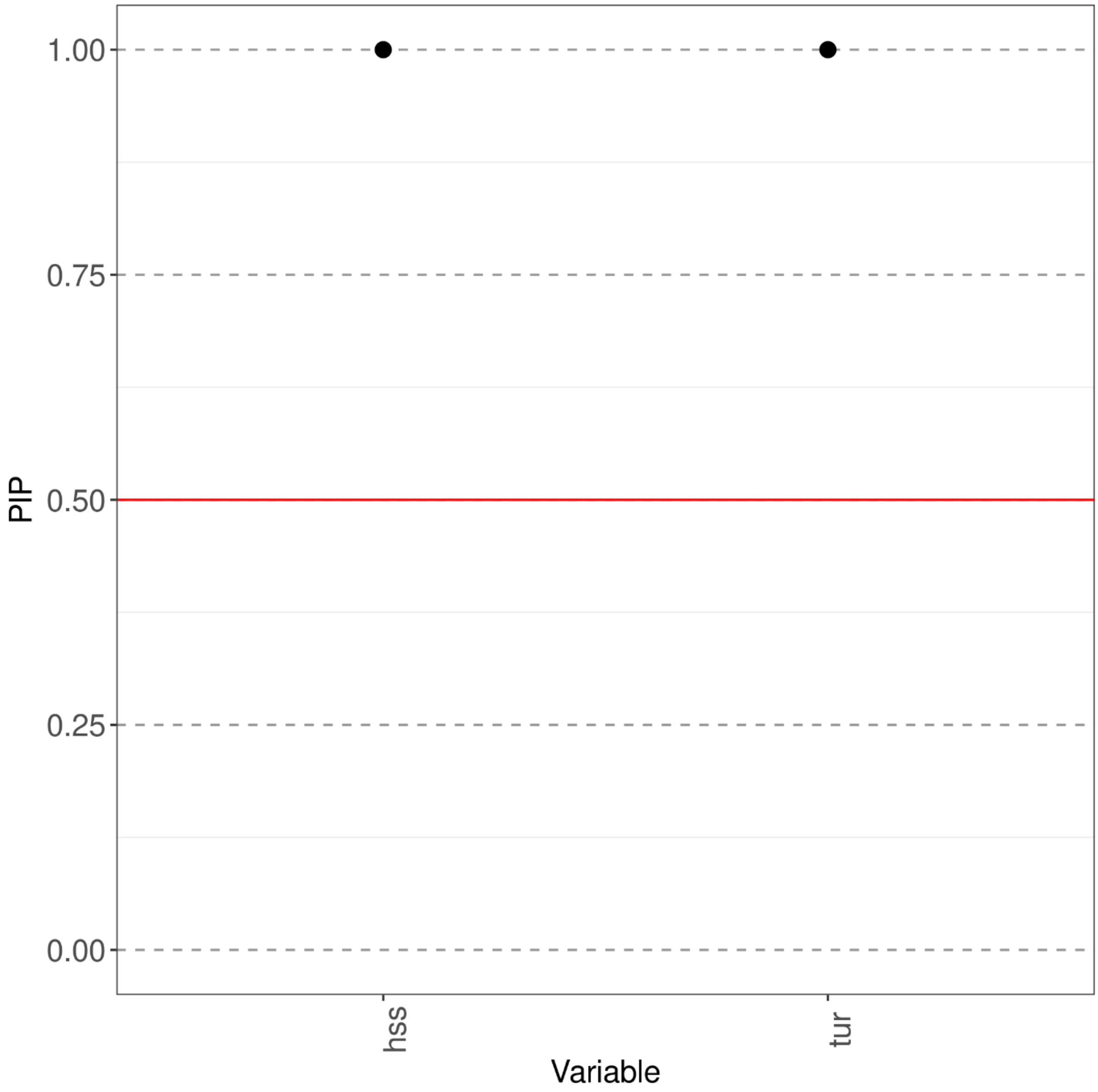

Figure S3. PIPs for the probability of occupancy. The horizontal line indicates the PIP = 0.5 line.

	0	1	2	3	4	5	6
1 - psi(x)	0.828443	0.828264	0.802788	0.349149	0.060072	0.047306	0.047172
q(x)	0.069193	0.026037	0.005467	0.013543	0.088598	0.318865	0.478297

Figure S4. First row: Posterior probability of species absence constrained by the number of positive qPCR replicates. Second row: posterior probability of a positive qPCR as a function of the presence of a species.

Table S1. eDNA concentration of samples from sites that was positive

Site	Sample replication	qPCR replication	eDNA concentration (copies/mL)	Mean	SD
**F**	1	1	531.3	531.3	8.79
		2	527.9		
		3	538.0		
		4	517.9		
		5	534.6		
		6	531.3		
	2	1	538.0		
		2	551.9		
		3	534.6		
		4	538.0		
		5	541.5		
		6	534.6		
	3	1	524.6		
		2	517.9		
		3	521.2		
		4	531.3		
		5	517.9		
		6	531.3		
**G**	1	1	4.8	4.7	0.42
		2	4.6		
		3	4.7		
		4	4.7		
		5	4.9		
		6	4.7		
	2	1	4.2		
		2	4.2		
		3	3.5		
		4	4.7		
		5	4.7		
		6	4.7		
	3	1	5.4		
		2	5.1		
		3	4.9		
		4	5.2		
		5	5.3		
		6	4.9		

Table S1. (cont.) eDNA concentration of samples from sites that was positive

Site	Sample replication	qPCR replication	eDNA concentration (copies/mL)	Mean	SD
**I**	1	1	470.9	458.4	10.15
		2	456.2		
		3	462.0		
		4	459.1		
		5	467.9		
		6	470.9		
	2	1	464.9		
		2	447.6		
		3	439.1		
		4	441.9		
		5	450.4		
		6	441.9		
	3	1	470.9		
		2	467.9		
		3	459.1		
		4	456.2		
		5	464.9		
		6	459.1		
**L**	1	1	5.4	5.3	0.15
		2	5.4		
		3	5.3		
		4	5.4		
		5	5.3		
		6	5.4		
	2	1	5.5		
		2	5.5		
		3	5.6		
		4	5.5		
		5	5.5		
		6	5.5		
	3	1	5.2		
		2	5.1		
		3	5.2		
		4	5.1		
		5	5.2		
		6	5.3		

Table S1. (cont.) eDNA concentration of samples from sites that was positive

Site	Sample replication	qPCR replication	eDNA concentration (copies/mL)	Mean	SD
**M**	1	1	3.3	3.3	0.11
		2	3.3		
		3	3.3		
		4	3.4		
		5	3.3		
		6	3.3		
	2	1	3.1		
		2	3.2		
		3	3.1		
		4	3.1		
		5	3.1		
		6	3.2		
	3	1	3.3		
		2	3.4		
		3	3.4		
		4	3.4		
		5	3.4		
		6	3.4		

Table S2. Accession number of COI sequences used in primers and probe design

Species	Accession number
Anabas testudineus	KX455903
Anematichthys repasson	KT001064
Anguilla bicolor	KF182304
Badis ruber	MK567726
Bagarius bagarius	KX455910
Bagarius suchus	DQ846698
Bagarius yarrelli	KM610421
Barbonymus altus	EF609294
Barbonymus gonionotus	KJ936769
Barbonymus schwanefeldii	MN342343
Betta smaragdina	GQ911870
Betta splendens	GQ911736
Botia rostrata	MK632322
Carassius auratus	JN673558
Carassius carassius	JQ319108
Carassius cuvieri	HQ536316
Carassius gibelio	JQ319082
Catlocarpio siamensis	HM536911
Channa gachua	KM272635
Channa lucius	KM213042
Channa marulioides	GQ334377
Channa melasoma	KJ937380
Channa micropeltes	KM213040
Channa striata	KC789519
Chanos chanos	DQ885083
Chitala blanci	AP008921
Chitala lopis	KM213054
Chitala ornata	EF609328
Cirrhina mrigala	KU559566
Cirrhinus cirrhosus	KT353104
Cirrhinus microlepis	HM536924
Cirrhinus molitorella	GU086576
Clarias batrachus	KC789524
Clarias fuscus	JN020071
Clarias gariepinus	KM261768
Clarias macrocephalus	MG407378
Ctenopharyngodon idella	JN673561
Cyprinus carpio	JN673560
Cyprinus rubrofuscus	KJ994623
Datnioides pulcher	KF753753
Datnioides undecimradiatus	KF753758
Datnioides undecimradiatus	KF753759

Table S2. (cont.) Accession number of COI sequences used in primers and probe design

Species	Accession number
Dermogenys siamensis	MG563401
Epalzeorhynchos bicolor	JF915594
Epalzeorhynchos frenatum	MN342579
Garra cambodgiensis	MN342588
Garra fasciacauda	JQ864618
Glossogobius aureus	KC789533
Hemibagrus microphthalmus	KJ909359
Hemibagrus nemurus	MN243484
Hemibagrus wyckii	JF781178
Henicorhynchus lobatus	MK116343
Henicorhynchus ornatipinnis	MK448107
Henicorhynchus siamensis	MK049365
Hypophthalmichthys molitrix	MH176327
Hypophthalmichthys nobilis	MH176328
Hypostomus plecostomus	MZ050829
Kryptopterus bicirrhis	KU568889
Kryptopterus cheveyi	MK049457
Kryptopterus geminus	MK448117
Labeo dyocheilus	KU207144
Labeo pierrei	KC631199
Labeo rohita	GU195112
Macrognathus siamensis	EF609404
Mastacembelus armatus	MK804146
Notopterus notopterus	KT022089
Ophiocara porocephala	MK572389
Oreochromis aureus	MH515227
Oreochromis mossambicus	MH515239
Oreochromis niloticus	MH515186
Oryzias mekongensis	MK156204
Osphronemus goramy	KU692699
Oxyeleotris marmorata	KT022088
Pangasianodon gigas	KY118584
Pangasianodon hypophthalmus	KX685193
Pangasius bocourti	EF609425
Pangasius conchophilus	KT289885
Pangasius djambal	KP036427
Pangasius krempfi	KT289877
Pangasius larnaudii	EU752152
Pangasius macronema	KT289888
Pangasius nasutus	KT001045
Pangasius pangasius	JX997836
Pangasius sanitwongsei	KC627282

Table S2. (cont.) Accession number of COI sequences used in primers and probe design

Species	Accession number
Parachela siamensis	MK049435
Phenacostethus smithi	AP006773
Probarbus jullieni	HM536909
Puntioplites bulu	MK621899
Puntioplites falcifer	HM536904
Puntioplites waandersi	KU692822
Puntius brevis	HM536914
Puntius chola	KJ936779
Puntius orphoides	JF915642
Puntigrus partipentazona	MT483480
Scaphiodonichthys acanthopterus	KJ994655
Scaphognathops bandanensis	HM536927
Scaphognathops stejnegeri	HM536906
Schistura poculi	KM610972
Schistura pridii	AP011443
Syncrossus helodes	JQ661349
Thynnichthys thynnoides	KC631204
Tor douronensis	JN646100
Tor putitora	KT762361
Tor sinensis	KJ994657
Tor tambroides	MW471072
Tor tor	KT200168
Toxotes chatareus	KY849559
Trichogaster microlepis	KU569058
Trichogaster pectoralis	HQ682726
Trichopsis schalleri	KP200391
Trigonostigma somphongsi	KX656878
Wallago attu	MK714085
Wallago leerii	MN992975
Wallago micropogon	MK448131
Yasuhikotakia modesta	JQ346170
Yasuhikotakia morleti	KU569084
